# Effect of High Pelvic Incidence on Fixation Failure in Single-Level Transforaminal Lumbar Interbody Fusion for Low-Grade Spondylolisthesis: A Retrospective Cohort Study

**DOI:** 10.3390/jcm15093199

**Published:** 2026-04-22

**Authors:** Koopong Siribumrungwong, Sansern Satthanan, Bunyaporn Wuttiworawanit, Punnawit Pinitchanon, Thongchai Suntharapa

**Affiliations:** 1 Chulabhorn International College of Medicine, Thammasat University, Pathum Thani 12120, Thailand; koopongs@gmail.com; 2 Department of Orthopedic, Rajthanee Nongkhae Hospital, Saraburi 18230, Thailand; sansern.satthanan@gmail.com; 3 Department of Clinical Science, School of Medicine, Walailak University, Nakhon Si Thammarat 80160, Thailand; 4 Department of Orthopedic, Faculty of Medicine, Thammasat University, Pathum Thani 12120, Thailand; boompunnawit@gmail.com (P.P.); suntharapa@yahoo.com (T.S.)

**Keywords:** spondylolisthesis, pelvic incidence, TLIF, pedicle screw loosening

## Abstract

**Objective:** To investigate the association between pelvic incidence (PI) and fixation failure following single-level transforaminal lumbar interbody fusion (TLIF) for low-grade spondylolisthesis and to identify risk factors for pedicle screw loosening. **Methods:** This retrospective study included 80 patients who underwent single-level TLIF and were divided into a high PI group (*n* = 40) and a normal/low PI group (*n* = 40). Radiographic parameters including PI, lumbar lordosis (LL), pelvic tilt (PT), sacra l slope (SS), listhesis magnitude, and PI-LL mismatch were evaluated pre- and postoperatively. Screw loosening and fusion status were assessed at 6, 12, and 24 months. Multivariate logistic regression analysis was performed to identify independent risk factors for screw loosening. **Results:** The high PI group demonstrated significantly higher screw loosening rates than the normal/low PI group at all follow-up time points, with a rate of 57.5% versus 28.2% at 24 months (*p* = 0.012). Fusion rates were comparable between groups. Multivariate analysis identified high PI and residual listhesis were independent risk factors for screw loosening (Odds ratio 1.05 and 1.35). PI-LL mismatch > 10° showed higher odds but were not statistically significant. **Conclusions:** High PI is associated with an increased risk of pedicle screw loosening after single-level TLIF. Careful preoperative assessment and postoperative monitoring may help reduce fixation-related complications.

## 1. Introduction

Spondylolisthesis is a common degenerative spinal disorder that frequently requires surgical intervention to achieve stabilization, neural decompression, and spinal fusion. Transforaminal lumbar interbody fusion (TLIF) combined with pedicle screw fixation is one of the most widely used surgical techniques for the treatment of low-grade spondylolisthesis due to its ability to provide circumferential fusion and restore sagittal alignment [1,2,3]. Despite its widespread use, pedicle screw loosening remains a significant mechanical complication following TLIF and may result in loss of reduction, delayed fusion, or construct failure [4].

Biomechanical studies have suggested that TLIF may be more susceptible to fixation failure compared with other interbody fusion techniques such as lateral lumbar interbody fusion (LLIF) and posterior lumbar interbody fusion (PLIF) of the. In particular, finite element analyses using L3–L5 models to simulate L4/5 fixation—the most affected level in degenerative spondylolisthesis—have demonstrated increased stress concentration on posterior instrumentation following TLIF [5]. However, clinical data identifying patient-related anatomical factors that predispose to fixation failure remain limited.

Spinopelvic alignment, particularly pelvic incidence (PI), plays a fundamental role in spinal biomechanics. Pelvic incidence is a fixed anatomical parameter that determines lumbar lordosis (LL), pelvic tilt (PT), and sacral slope (SS). A high PI has been associated and has been implicated in the development and progression of spondylolisthesis [6]. Based on this biomechanical concept, we hypothesized that patients with a high PI may present with a greater degree of listhesis, require increased mechanical demand for reduction, and exhibit a higher likelihood of residual or progressive listhesis after surgery compared with patients with a normal PI. In addition, inadequate restoration of spinopelvic alignment or persistent postoperative sagittal imbalance, such as PI-LL mismatch, or incomplete reduction [7] may further increase mechanical stress on the instrumentation. These factors may collectively contribute to an increased risk of screw loosening or implant failure. While PI-LL mismatch has been extensively studied as a predictor of sagittal imbalance and clinical outcomes, the direct relationship between pelvic incidence and implant-related complications [8,9,10], such as pedicle screw loosening after short-segment fusion, has not been fully elucidated. Unlike previous studies focusing on long-segment fusion or global malalignment, this study specifically isolates the effect of intrinsic pelvic morphology on fixation stability in short-segment TLIF.

Therefore, the purpose of this study was to evaluate the radiographic outcomes, fusion rates, and pedicle screw loosening following single-level TLIF for low-grade spondylolisthesis in patients with high versus normal/low pelvic incidence. We further aimed to identify independent risk factors associated with screw loosening, with particular emphasis on the role of pelvic incidence and postoperative alignment parameters.

## 2. Materials and Methods

### 2.1. Patient Selection

This retrospective cohort study included 80 patients (40 with high pelvic incidence (PI) and 40 with normal/low PI) who underwent single-level transforaminal lumbar interbody fusion (TLIF) for low-grade spondylolisthesis at Thammasat University Hospital between 1 January 2020, and 1 January 2022. The study was conducted in accordance with the Declaration of Helsinki and was approved by the Human Ethics Committee of Thammasat University (approval No. MTU-EC-OT-0-077/67) on 7 May 2024. Patients lacking demographic data or postoperative radiographs at 3, 6, 12, and 24 months were excluded from the study.

### 2.2. Data Collection

Electronic medical records were reviewed to collect demographic, surgical, and radiographic data. High PI was defined as a pelvic incidence greater than 55°. Demographic variables included age, sex, and bone mineral density (BMD). Osteoporosis was defined as a BMD T-score less than −2.5. Surgical characteristics included operative level (L4/5 or L5/S1) and surgical approach (open or minimally invasive). The choice of surgical approach was determined through shared decision-making between the surgeon and the patient, considering patient preference, economic factors, and anatomical complexity. Both techniques used the same fusion principles and instrumentation.

Radiographic parameters were measured independently by two independent reviewers. Preoperative radiographic parameters included pelvic incidence (PI), lumbar lordosis (LL), pelvic tilt (PT), sacral slope (SS), and the degree of spondylolisthesis measured on standing lateral radiographs. Postoperative radiographic parameters (LL, PT, SS, and degree of spondylolisthesis) were assessed at the immediate postoperative period and at 3, 6, 12, and 24 months following surgery. The PI-LL mismatch was calculated as the numerical difference between PI and LL.

Screw loosening was defined as the presence of a radiolucent line greater than 1 mm around the pedicle screw, screw displacement, pullout, or mechanical failure on follow-up radiographs. Fusion at the L5/S1 level was evaluated using anteroposterior and lateral plain radiographs according to the grading system described by Patil et al., with successful fusion defined as grade 2 or higher [11].

All TLIF procedures were performed by single experienced spine surgeons using standard surgical techniques. The choice of surgical approach (open or minimally invasive) and instrumentation was based on surgeon preference and patient-specific factors. All patients followed a standardized postoperative rehabilitation protocol, including early mobilization and physical therapy.

The primary outcome was the incidence of pedicle screw loosening. Secondary outcomes included fusion rate, postoperative reduction and progression of listhesis, postoperative PI-LL mismatch, and identification of risk factors associated with screw loosening at a minimum follow-up of 24 months.

### 2.3. Statistical Analysis

Statistical analysis was performed using Stata version 16 (Stata Corp., College Station, TX, USA). Descriptive statistics were used to summarize patient characteristics and radiographic parameters. Continuous variables were expressed as mean ± standard deviation or median with interquartile range, while categorical variables were presented as frequencies and percentages. Between-group comparisons were performed using Student’s *t*-test or chi-square test, as appropriate.

Multivariate logistic regression analysis was conducted using both continuous and categorical variables to identify independent risk factors for screw loosening. The variables included postoperative listhesis greater than 3 mm (as residual listhesis > 3 mm has been reported to be associated with screw loosening [10]), postoperative PI-LL mismatch greater than 10° (based on the sagittal modifier of the SRS-Schwab classification [12]), high pelvic incidence (defined as PI > 55°, representing the transitional range toward high pelvic incidence as previously reported [7]), and osteoporosis. A *p*-value of less than 0.05 was considered statistically significant.

## 3. Results

### 3.1. Characteristics of the of Study Population

A total of 80 patients were included in the study, with 40 patients in the high pelvic incidence (PI) group and 40 patients in the normal/low PI group. There were no significant differences between the two groups in age, bone mineral density (BMD), prevalence of osteoporosis, surgical level (L4/5 vs. L5/S1), or surgical approach (open vs. minimally invasive). However, a significantly higher proportion of female patients was observed in the high PI group (*p* = 0.01).

Preoperative radiographic parameters differed significantly between groups. The high PI group demonstrated greater lumbar lordosis, pelvic tilt, sacral slope, and preoperative listhesis compared with the normal/low PI group (all *p* < 0.001) (Table 1).

### 3.2. Radiographic Outcome and Complications

Lumbar lordosis, pelvic tilt, and sacral slope remained significantly higher in the high PI group at all postoperative time points, including the immediate postoperative period and up to 24 months of follow-up (all *p* < 0.001). These findings indicate persistent differences in spinopelvic alignment between groups despite surgical correction. The PI-LL mismatch rate was higher in the high PI group at the 24-month postoperative follow-up (26 patients [65%] vs. 16 patients [40%] in the normal/low PI group); however, the difference did not reach statistical significance (Table 2).

Interobserver reliability of radiographic measurements was assessed across all follow-up time points (Table A2). Overall, reliability was good to excellent for all parameters. Lumbar lordosis, listhesis, and PI-LL mismatch demonstrated excellent agreement across most time points (ICC > 0.90), whereas pelvic incidence, pelvic tilt, and sacral slope showed good to excellent reliability (ICC range 0.85–0.91).

**Figure 1:**
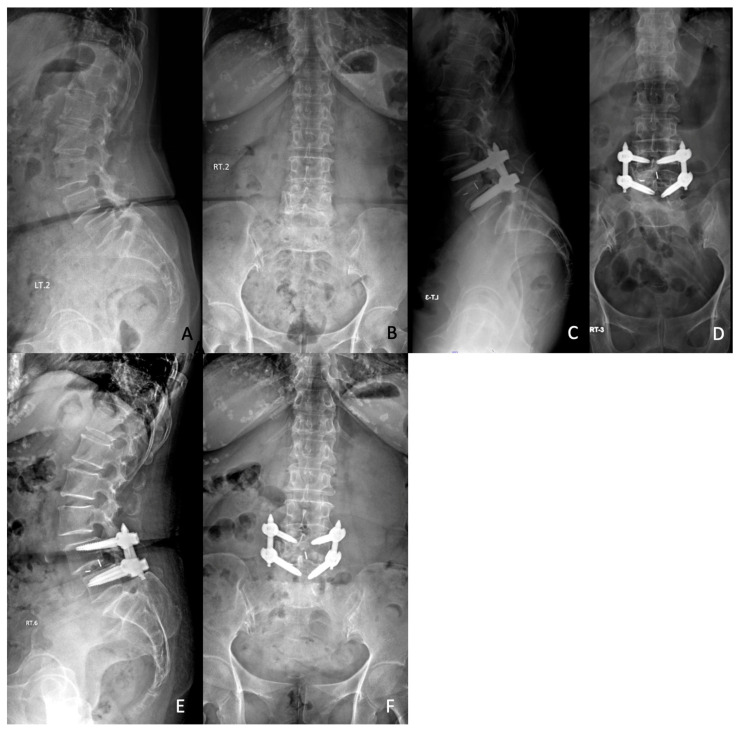
Representative case of fixation failure in a 71-year-old female with high pelvic incidence. (**A**,**B**) Preoperative standing radiographs demonstrate a high PI (71°) with degenerative spondylolisthesis. (**C**,**D**) Immediate postoperative images show satisfactory reduction and restoration of lumbar lordosis, with improved sagittal alignment. (**E**,**F**) At 6 months postoperatively, radiographs reveal screw loosening with loss of reduction and recurrent listhesis (7.53 mm).

Screw loosening rate was higher in the high PI group at all postoperative follow-up intervals. Although no statistically significant difference was found at 3 months postoperatively (35% vs. 23%, *p* = 0.32), the incidence of screw loosening was significantly higher in the high PI group at 6 months (55% vs. 28.3%, *p* = 0.03), 12 months (57.5% vs. 28.2%, *p* = 0.012), and 24 months (57.5% vs. 28.2%, *p* = 0.012) (Table 3) (Figure 1).

As shown in Table 4, multivariable logistic regression analysis demonstrated that higher pelvic incidence (PI) was significantly associated with screw loosening (OR 1.05, 95% CI 1.01–1.10, *p* = 0.034) in Model 1, whereas bone mineral density (BMD) was not a significant predictor. In Model 2, postoperative listhesis was identified as an independent risk factor for screw loosening (OR 1.35, 95% CI 1.04–1.81, *p* = 0.030), while postoperative PI-LL mismatch was not significantly associated with the outcome (OR 1.04, 95% CI 0.98–1.10, *p* = 0.227), although the direction of effect suggested a potential positive association. Separate models were constructed to minimize multicollinearity among spinopelvic parameters.

Consistent with these findings, categorical analysis using predefined thresholds (Table A1) demonstrated that high PI (PI > 55°) was the only independent factor significantly associated with screw loosening (OR 3.06, 95% CI 1.17–8.40, *p* = 0.03). In contrast, postoperative listhesis greater than 3 mm (OR 2.07, *p* = 0.27) and PI-LL mismatch greater than 10° (OR 1.17, *p* = 0.75) were not statistically significant, although their direction of effect suggested a potential increase in risk. Osteoporosis was also not significantly associated with screw loosening.

## 4. Discussion

The primary finding of this study is that a high pelvic incidence is significantly associated with increased rates of pedicle screw loosening following single-level TLIF for low-grade spondylolisthesis. At the 24-month follow-up, the loosening rate in the high PI group was nearly double that of the normal/low PI group (57.5% vs. 28.2%). Importantly, multivariate analysis identified high PI as the only statistically significant independent factor within this cohort.

High pelvic incidence has been widely studied in the context of adult spinal deformity (ASD), particularly in long-segment fusion. Ushio et al. reported that high PI was a significant risk factor for lower instrumented vertebra (LIV) failure after long corrective fusion terminating at L5 [8]. Conversely, Banno et al. found no difference in PI between loosening and non-loosening groups but identified poor postoperative alignment parameters, including pelvic tilt, thoracic pelvic angle, and PI-LL mismatch, as risk factors [9]. Although these studies involved long-segment fusion and differed in methodology, our findings suggest that even in short-segment fusion such as single-level TLIF, intrinsic pelvic morphology—specifically high PI—plays a critical role in implant stability.

The association between high pelvic incidence (PI) and screw loosening observed in our study may be explained by altered biomechanical loading. According to Roussouly et al., a high PI is associated with increased lumbar lordosis and sacral slope, resulting in greater anterior shear forces across the L5–S1 disc and increased traction across the pars interarticularis [5,6]. In patients with high PI, this augmented shear loading is transmitted to the pedicle screw–rod construct following TLIF, leading to increased micromotion at the bone–screw interface and predisposing to screw loosening. In addition, patients in the high PI group demonstrated greater preoperative listhesis and higher residual listhesis during follow-up, both of which may further amplify mechanical stress on the instrumentation [5]. Collectively, these biomechanical factors may contribute to an increased risk of implant instability and screw loosening in this patient population.

This highlights the importance of careful preoperative planning to prevent screw loosening in patients with high pelvic incidence (PI), given its potential impact on clinical outcomes as reported in previous studies [13].

Our results regarding screw loosening are consistent with biomechanical principles and prior clinical studies. Kim et al. investigated risk factors for S1 screw loosening following TLIF and identified high PI, greater postoperative lumbar lordosis change, and larger PI-LL mismatch as significant contributors to screw loosening, in addition to age, bone mineral density, and multilevel fusion [14]. Our results are partially consistent with these findings; however, multivariate logistic regression analysis in the present study did not demonstrate a significant association between osteoporosis and screw loosening. This discrepancy from previous reports may be attributable to the relatively low prevalence of osteoporotic patients in our cohort, which may have limited the statistical power to detect an independent effect of bone mineral density on implant stability.

Although the clinical outcome was not included in the data collection in our study, indirectly, we found that the factors that influence the clinical outcome were different between groups. The factors included PI-LL mismatch and the residual listhesis. The listhesis was significantly more pronounced in the high PI group at 3, 6, 12, and 24 months postoperatively. Bokov et al. reported that residual listhesis greater than 3 mm, incomplete reduction was associated with an increased risk of screw loosening in degenerative lumbar disease, and 39 out of 97 patients with screw loosening complained of axial back pain with ODI of more than 40 and required revision surgery [10].

Similarly, regarding PI-LL mismatch, several previous studies have demonstrated that PI-LL mismatch is an important factor associated with screw loosening and has a significant impact on clinical outcomes, particularly in long-construct surgery for patients with degenerative scoliosis or sagittal imbalance [8,9]. Consistently, Cui et al. identified PI-LL mismatch as one of the key predictors of unfavorable outcomes in short-segment constructs [15] which is in agreement with the findings of Aoki et al., who investigated outcomes in patients undergoing short-segment TLIF. In their study, patients with PI-LL mismatch (PI-LL ≥ 11°) demonstrated poorer postoperative visual analog scale (VAS) scores for back pain and low back pain in standing position, and showed a statistically significant association with postoperative numbness at 1-year follow-up. However, no significant difference in Oswestry Disability Index (ODI) scores was observed between the groups [16,17]. Beyond clinical outcomes, Takeda et al. demonstrated that both preoperative and postoperative PI-LL mismatch (≥15°) are significant risk factors for early-onset radiographic adjacent segment disease (ASD) in patients with spondylolytic spondylolisthesis undergoing single-level TLIF. In their study, ASD developed at a mean duration of 21.7 ± 12.6 months following the initial surgery [17].

In contrast, a recent systematic review and meta-analysis by Bryan Liyis et al. reported that pelvic incidence (PI) and PI-LL mismatch were not significant predictors of screw loosening [18]. We speculate that this discrepancy may be attributed to differences in patient populations across the included studies. Notably, the mean PI values in those studies were within the range corresponding to the low PI group based on our cut-off value. This suggests a relatively low proportion of high PI patients in their cohorts, which may have attenuated the observed effect of PI on screw loosening. Another large cohort by Srikanth et al. [19] also reported PI-LL mismatch (PI-LL > 10) did not affect the clinical outcome which may be explained by several factors beyond the distribution of pelvic incidence. First, in short-segment constructs, global sagittal alignment may play a less dominant role compared to long-segment deformity correction, as the biomechanical demand is more localized. Second, PI-LL mismatch is a global alignment parameter and may not fully reflect segmental biomechanics at the fused level. Local factors such as residual listhesis, segmental instability, and disc degeneration may exert a greater influence on mechanical loading and implant behavior in short-segment TLIF. Finally, compensatory mechanisms, including pelvic tilt and adjacent segment adaptation, may mitigate the clinical impact of PI-LL mismatch, thereby weakening its association with clinical outcomes. Therefore, the role of PI-LL mismatch as an independent predictor in short-segment fusion remains controversial. This may also explain why, in our study, PI-LL mismatch demonstrated only a trend toward increased risk without reaching statistical significance.

However, radiographic parameters alone may not fully explain postoperative outcomes. A recent study by Łosiński et al., using three-dimensional posturography, demonstrated that postoperative spinal alignment is dynamic and influenced by functional and diurnal factors, which are not fully captured by static radiographic assessments. This may partly explain why radiographic findings do not always correlate with mechanical outcomes [20].

With respect to fusion outcomes, our study demonstrated a significantly lower fusion rate in the high PI group at 6 months postoperatively. However, this difference diminished over time, and by 24 months, fusion rates were comparable between groups (97.5% vs. 100%). These findings suggest that high PI may be associated with delayed fusion rather than ultimate nonunion. This observation is consistent with previous reports demonstrating high long-term fusion rates following TLIF, including the study by Lv et al., which reported a fusion rate of 95.8% following minimally invasive TLIF [21].

From a clinical perspective, our findings highlight the importance of comprehensive preoperative assessment of spinopelvic parameters. High PI should be considered when planning reduction and fixation strategy in short-segment TLIF. Surgeons should carefully evaluate pelvic incidence, degree of listhesis, and lumbar lordosis to optimize surgical planning and minimize the risk of mechanical complications. In such cases, patients may benefit from more robust fixation strategies, including the use of larger-diameter screws, bicortical fixation, cement augmentation, or the addition of supplemental fixation points to enhance construct stability and reduce the risk of screw loosening. Based on our findings, reduction of the listhesis to less than 3 mm may help mitigate the risk of screw loosening, in accordance with previous studies [10,14].

Bone mineral density is another well-established risk factor for screw loosening. Although osteoporosis was not significantly associated with screw loosening in our study, this finding should be interpreted with caution due to the small number of osteoporotic patients included. Prior studies have consistently demonstrated an association between low BMD and implant failure [14,22], and BMD remains an important consideration in surgical decision-making.

Several limitations should be acknowledged. First, the retrospective design may have introduced selection bias and unmeasured confounding factors. Second, the relatively small sample size limits statistical power, may contribute to wide confidence intervals, and reduces the generalizability of the findings. Third, this study lacked clinical outcome data, including validated patient-reported outcome measures such as the Oswestry Disability Index (ODI), which precluded assessment of the clinical relevance of radiographic findings. In addition, fusion status was assessed using plain radiographs rather than computed tomography, which may have reduced the accuracy of fusion evaluation and underestimated nonunion.

Future research should focus on prospective studies with larger sample sizes, longer follow-up periods, and incorporation of comprehensive clinical outcome measures. In particular, integrating radiographic parameters with functional and biomechanical data may enable more precise risk stratification and support individualized surgical planning, thereby improving the understanding of the multifactorial mechanisms underlying pedicle screw loosening [23,24,25].

## 5. Conclusions

In conclusion, high pelvic incidence (PI > 55°) appears to be an independent risk factor for pedicle screw loosening following single-level TLIF in patients with low-grade spondylolisthesis. While long-term fusion rates remain satisfactory regardless of PI values, patients with high PI tended to show more susceptible to early fixation failure and a subsequent loss of surgical reduction. These findings suggest that a “one-size-fits-all” approach may not be appropriate for TLIF; instead, preoperative spinopelvic parameters should be carefully evaluated. For patients identified with high PI, surgeons should consider enhanced fixation strategies or more meticulous sagittal balance restoration to counteract the heightened mechanical demands and ensure long-term structural stability.

## Figures and Tables

**Table 1:** Comparison of Patient Characteristics Between Normal/Low and High PI Groups.

Demographic Data	High PI	Normal/Low PI	*p*-Value
Age (mean: SD)	61 (8.09)	62 (9.64)	0.65
Sex (Male:Female)	(3:37)	(12:27)	0.01 *
BMD (mean: SD)	−1.2 (1.04)	−1.33 (0.93)	0.76
Osteoporosis (BMD < −2.5)	3 (7.5%)	5 (12.5%)	0.13
Level - L4/5 - L5/S1			1.00
	34 (85)	34 (85)	
	6 (15)	6 (15)	
Surgery - Open - MIS			0.65
	20 (50%)	23 (57.5%)	
	20 (50%)	17 (42.5%)	
PI (mean: SD)	69 ± 4.1	51 ± 6.6	<0.001 *
Pre-op LL (degree) (mean: SD)	54 ± 11.2	43 ± 13	<0.001 *
Pre-op PT (degree) (mean: SD)	26 ± 7.5	20 ± 6.2	<0.001 *
Pre-op SS (degree) (mean: SD)	42 ± 8.06	31 ± 7.2	<0.001 *
Pre-op listhesis (mm) (mean: SD)	5.8 ± 3.65	3.1 ± 2.5	<0.001 *

SD, Standard deviation; PI, pelvic incidence; BMD, bone mineral density. * *p* < 0.05.

**Table 2:** Comparison of Preoperative and Postoperative Parameters Between Normal/Low and High PI Groups.

	Preop	Immediate	3 Months	6 Months	12 Months	24 Months
Listhesis						
- High PI	5.8 ± 3.6	1.8 ± 2.1	3.2 ± 3.0	3.8 ± 3.3	4.1 ± 3.4	4.2 ± 3.5
- Low PI	3.1 ± 2.5	0.8 ± 1.4	1.2 ± 2.1	1.5 ± 2.7	1.5 ± 2.7	1.5 ± 2.7
*p*-value	<0.001 *	0.11	0.001 *	<0.001 *	<0.001 *	<0.001 *
LL						
- High	54 ± 11.2	54 ± 8.8	55 ± 8.7	56 ± 8.4	56 ± 8.9	56 ± 8.5
- Low	43 ± 13	44 ± 10.1	44 ± 10.4	44 ± 10.3	44 ± 10.3	44 ± 10.3
*p*-value	<0.001 *	<0.001 *	<0.001 *	<0.001 *	<0.001 *	<0.001 *
PT						
- High PI	26 ± 7.5	26 ± 7.6	28 ± 5.2	27 ± 4.9	28 ± 4.8	28 ± 4.9
- Low PI	20 ± 6.2	16 ± 7.2	20 ± 7.6	20 ±7.6	20 ± 8.0	20 ± 8.0
*p*-value	<0.001 *	<0.001 *	<0.001 *	<0.001 *	<0.001 *	<0.001 *
SS						
- High PI	42 ± 8.06	43 ± 7.8	40 ± 5.8	41 ± 5.6	41 ± 5.6	41 ± 5.6
- Low PI	31 ± 7.2	35 ± 6.9	30 ± 6.7	30 ± 6.5	30 ± 6.6	30 ± 6.6
*p*-value	<0.001 *	<0.001 *	<0.001 *	<0.001 *	<0.001 *	<0.001 *
PI-LL mismatch (>10°)						
- High PI	23 (57.5%)	26 (65.0%)				26 (65.0%)
- Low PI	13(32.5%)	14 (35.0%)				16 (40.0%)
*p*-value	0.54	0.02 *				0.06

PI, pelvic incidence; LL, lumbar lordosis; PT, pelvic tilt; SS, sacral slope; PI-LL, mismatch of pelvic incidence and lumbar lordosis. * *p* < 0.05.

**Table 3:** Comparison of Complications Between Normal/Low and High PI Groups.

	3 Months	6 Months	12 Month	24 Months
Fusion rate				
- High PI		7 (17.5)	36 (90.0)	39 (97.5)
- Low PI		28 (71.79)	40 (100)	40 (100)
*p*-value		<0.001 *	0.11	0.62
Screw loosening				
- High PI	14 (35%)	22 (55%)	23 (57.5%)	23 (57.5%)
- Low PI	9 (23%)	11 (28.3%)	11 (28.2%)	11 (28.2%)
*p*-value	0.32	0.03 *	0.012 *	0.012 *

PI, pelvic incidence. * *p* < 0.05.

**Table 4:** Multivariable logistic regression analysis of factors associated with screw loosening.

Variable	Model 1 (PI and BMD) OR (95% CI)	*p*-Value	Model 2 (Residual Listhesis and PI-LL Mismatch) OR (95% CI)	*p*-Value
PI (per 1° increase)	1.05 (1.01–1.10)	0.034 *	–	–
BMD (T-score)	1.14 (0.71–1.84)	0.590	–	–
Listhesis (mm)	–	–	1.35 (1.04–1.81)	0.030 *
PI-LL mismatch (°)	–	–	1.04 (0.98–1.10)	0.227

95% CI, 95% confidence interval; OR, odds ratio; mm., millimeters; PI, pelvic incidence; PI-LL, mismatch of pelvic incidence and lumbar lordosis; BMD, bone mineral density. * *p* < 0.05.

## Data Availability

The datasets generated and/or analyzed during the current study are not publicly available due to ethical considerations. However, the minimal dataset is available from the co-corresponding author upon reasonable request.

## Abbreviations

The following abbreviations are used in this manuscript:

PI	Pelvic incidence
TLIF	Transforaminal lumbar interbody fusion
LL	Lumbar lordosis
PT	Pelvic tilt
SS	Sacral slope
OR	Odds ratio
BMD	Bone mineral density
CI	Confidence interval

## Appendix A

**Table A1:** Multiple regression logistic analysis of possible risk factors for screw loosening.

Factors	Logistic Regression		
	*p*-Value	Odds Ratio	95% CI
Postoperative listhesis > 3 mm.	0.27	2.07	0.58, 7.96
Postoperative PI-LL mismatch > 10 mm.	0.75	1.17	0.44, 3.11
High PI (PI > 55 degrees)	0.03 *	3.06	1.17, 8.40
Osteoporosis (BMD < −2.5)	0.81	0.82	0.15, 4.00

95% CI, 95% confidence interval; mm., millimeters; PI, pelvic incidence; PI-LL, mismatch of pelvic incidence and lumbar lordosis; BMD, bone mineral density. * *p* < 0.05.

**Table A2:** Interobserver reliability of radiographic measurements at each follow-up time point.

Parameter	ICC (95% CI)					
	Preop	Immediate	3 Months	6 Months	12 Month	24 Months
PI (°)	0.88 (0.82–0.92)	0.87 (0.81–0.91)	0.89 (0.84–0.93)	0.88 (0.82–0.92)	0.90 (0.85–0.94)	0.91 (0.86–0.95)
LL (°)	0.93 (0.89–0.96)	0.92 (0.87–0.95)	0.94 (0.90–0.97)	0.93 (0.89–0.96)	0.95 (0.91–0.97)	0.96 (0.92–0.98)
PT (°)	0.86 (0.79–0.91)	0.85 (0.78–0.90)	0.87 (0.81–0.91)	0.86 (0.79–0.91)	0.88 (0.82–0.92)	0.89 (0.83–0.93)
SS (°)	0.87 (0.81–0.91)	0.86 (0.79–0.91)	0.88 (0.82–0.92)	0.87 (0.81–0.91)	0.89 (0.83–0.93)	0.90 (0.85–0.94)
Listhesis (mm)	0.91 (0.86–0.95)	0.90 (0.85–0.94)	0.92 (0.88–0.96)	0.91 (0.86–0.95)	0.93 (0.89–0.96)	0.94 (0.90–0.97)
PI-LL mismatch (°)	0.92 (0.88–0.96)	0.91 (0.86–0.95)				0.95 (0.91–0.98)

ICC, intraclass correlation coefficient; 95% CI, 95% confidence interval; mm., millimeters; PI, pelvic incidence; PI-LL, mismatch of pelvic incidence and lumbar lordosis. CC values were interpreted as follows: <0.5, poor; 0.5–0.75, moderate; 0.75–0.9, good; >0.9, excellent.
